# Supplementary material for: p53 Is Regulated in a Biphasic Manner in Hypoxic Human Papillomavirus Type 16 (HPV16)-Positive Cervical Cancer Cells
Source: Int J Mol Sci. 2020 Dec 15;21(24):9533. doi: 10.3390/ijms21249533 (PMC7765197; doi:10.3390/ijms21249533)
Supplement: Supplementary file 1 [file ijms-21-09533-s001.pdf]

Supplementary Material

Table 1. Quantification of p53 band intensities normalized to GAPDH from three independent experiments displayed in Figure 1C. Time indicates hours under hypoxia, respectively. *** $p < 0.001$, ** $p < 0.01$, * p < 0.0,5, ns: not significant.

Time (h)	SiHa #1	SiHa #2	SiHa #3	Average	SD	*p* Value	Stat
0	1.000	1.000	1.000	1.000	0.000		
6	0.776	0.802	0.982	0.854	0.112	0.152	ns
24	0.009	0.003	0.123	0.045	0.068	0.002	**
48	0.990	0.517	0.461	0.656	0.290	0.177	ns
72	1.939	2.786	2.254	2.326	0.428	0.033	*
**Time (h)**	**CaSki #1**	**CaSki #2**	**CaSki #3**	**Average**	**SD**	***p* Value**	**Stat**
0	1.000	1.000	1.000	1.000	0.000		
6	0.316	0.018	0.005	0.113	0.176	0.013	*
24	0.045	0.052	0.022	0.040	0.016	0.000	***
48	0.224	0.390	0.201	0.272	0.103	0.007	**
72	0.776	0.662	0.606	0.681	0.086	0.024	*

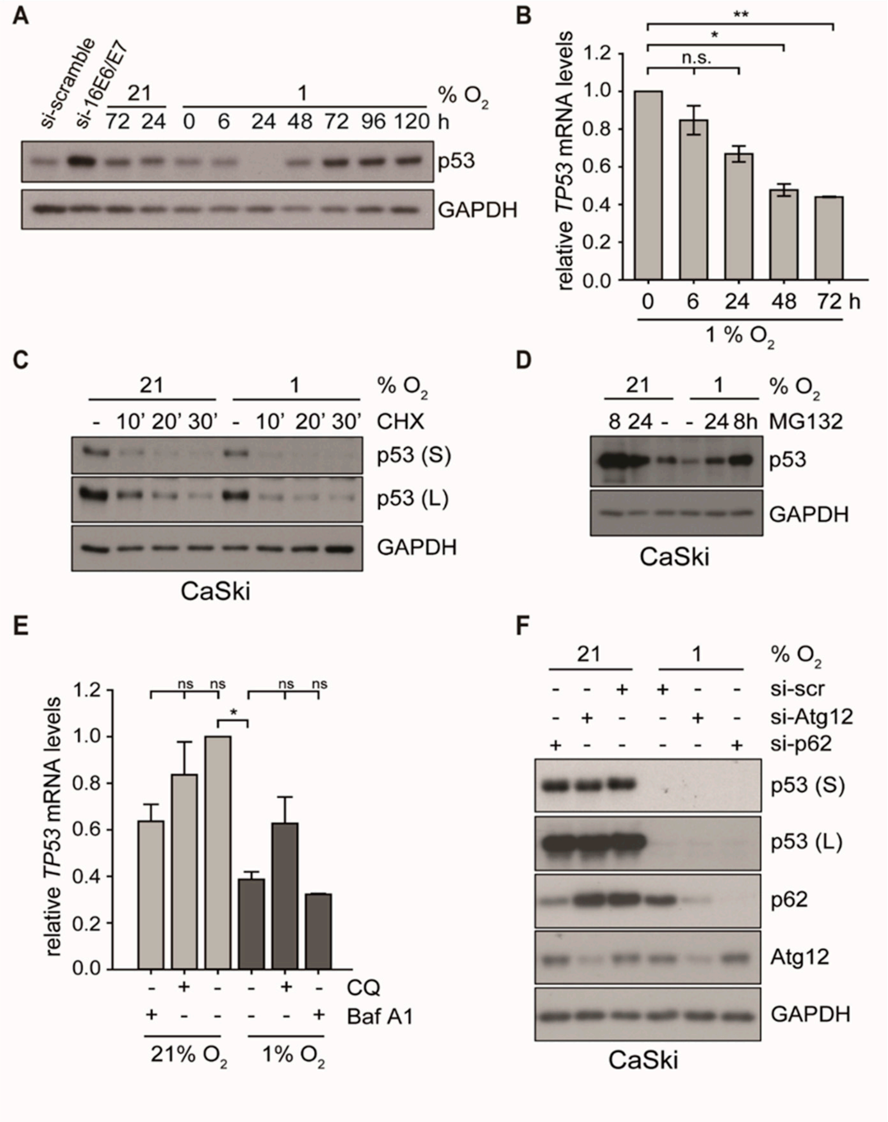

Figure S1. p53 transcript levels and protein stability in HPV16-positive cells, effect of lysosomal inhibitors on *TP53* transcript levels, and effect of silencing of *Atg12* or *p62* on p53. (**A**) HPV16 *E6*/*E7* silencing under normoxia in SiHa cells (si-16E6E7). Total protein was extracted 48 h after transfection. In parallel, cells were cultured for the indicated times under normoxia (21% O_2) or hypoxia (1% O_2) prior to protein extraction. Western blot analyses show levels of p53. GAPDH served as a loading control. (**B**) CaSki cells were cultured for the indicated times at 1% O_2. Total RNA was extracted, and *TP53* transcript levels were quantified by qPCR. The *18S* rRNA served as internal control. Error bars indicate the SD of three independent experiments. The 0 h value of *TP53* expression was set as a control for the calculation of significant differences (two-tailed Student's t-test was employed, * $p < 0.05$, ** $p < 0.01$ and ns, statistically not significant). (**C**) CaSki cells were incubated at 21% O_2 or at 1% O_2, respectively, for 2 h and subsequently treated with/without cycloheximide (CHX, 10 μg/mL) for up to 30 min prior to protein extraction. Western Bbot analyses using antibody against p53. GAPDH served as a loading control (S, short exposure; L, long exposure). (**D**) Western blot analyses using antibody against p53. CaSki cells were incubated at 21% O_2 or at 1% O_2 for 0 h or 16 h prior to the treatment with 10 μM MG132. Total protein was extracted after 24 h. GAPDH served as a loading control. (**E**) qPCR analyses of *TP53* levels. SiHa cells were cultivated for 24 h under normoxia (21% O_2) or hypoxia (1% O_2), in either the absence (−) or the presence (+) of 50 μM chloroquine (CQ) or 0.5 μM bafilomycin A1 (Baf A1) prior to RNA extraction. The *18S* rRNA served as internal control. Respective transcript levels of normoxic control samples were set to 1. Error bars indicate the SD of three independent experiments (for calculation of significant differences vs. respective normoxic control or hypoxic control, two-tailed Student's t-test was employed, * $p < 0.05$ and ns, statistically not significant). (**F**) Western blot analyses using antibodies against p53, Atg12, and p62. CaSki cells were transfected with scrambled control, *Atg12*-, or *p62*-specific siRNA. At

48 h post transfection, the cells were cultured for 24 h at 21% O_2 or at 1% O_2 prior to protein extraction. GAPDH served as a loading control (S, short exposure; L, long exposure).

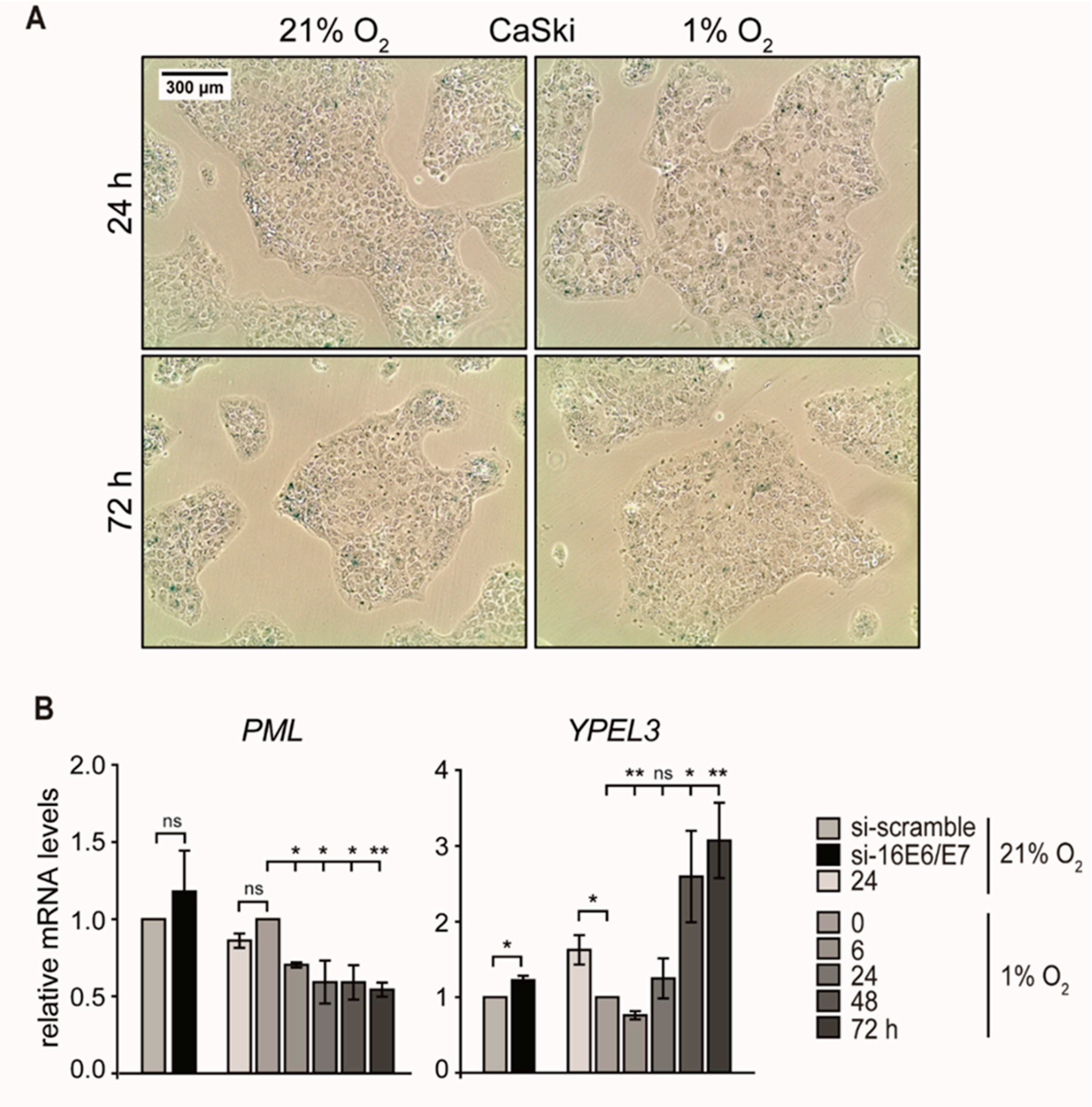

Figure S2. RNAi-mediated repression of HPV16 *E6/E7* and hypoxia modulate senescence in HPV16-positive CaSki cells. (**A**) CaSki cells were cultured under normoxia (21% O_2) or hypoxia (1% O_2). After 24 h or 72 h, cells were stained for the expression of SA-β-Gal. Scale bar: 300 μm. (**B**) HPV16 *E6/E7* expression was silenced by RNAi under normoxia in CaSki cells (si-16E6/E7), and total RNA was extracted 48 h after transfection. In parallel, cells were cultured for the indicated periods under normoxia (21% O_2) or hypoxia (1% O_2), and total RNA was extracted after respective time intervals. The *18S* rRNA served as internal control. Error bars indicate the SD of three independent experiments. The si-scramble group or the 0 h hypoxia group of gene expression was arbitrarily set to 1 in order to calculate significant differences (two-tailed Student's t-test was employed, ** $p < 0.01$, * $p < 0.05$ and ns, no significant difference).

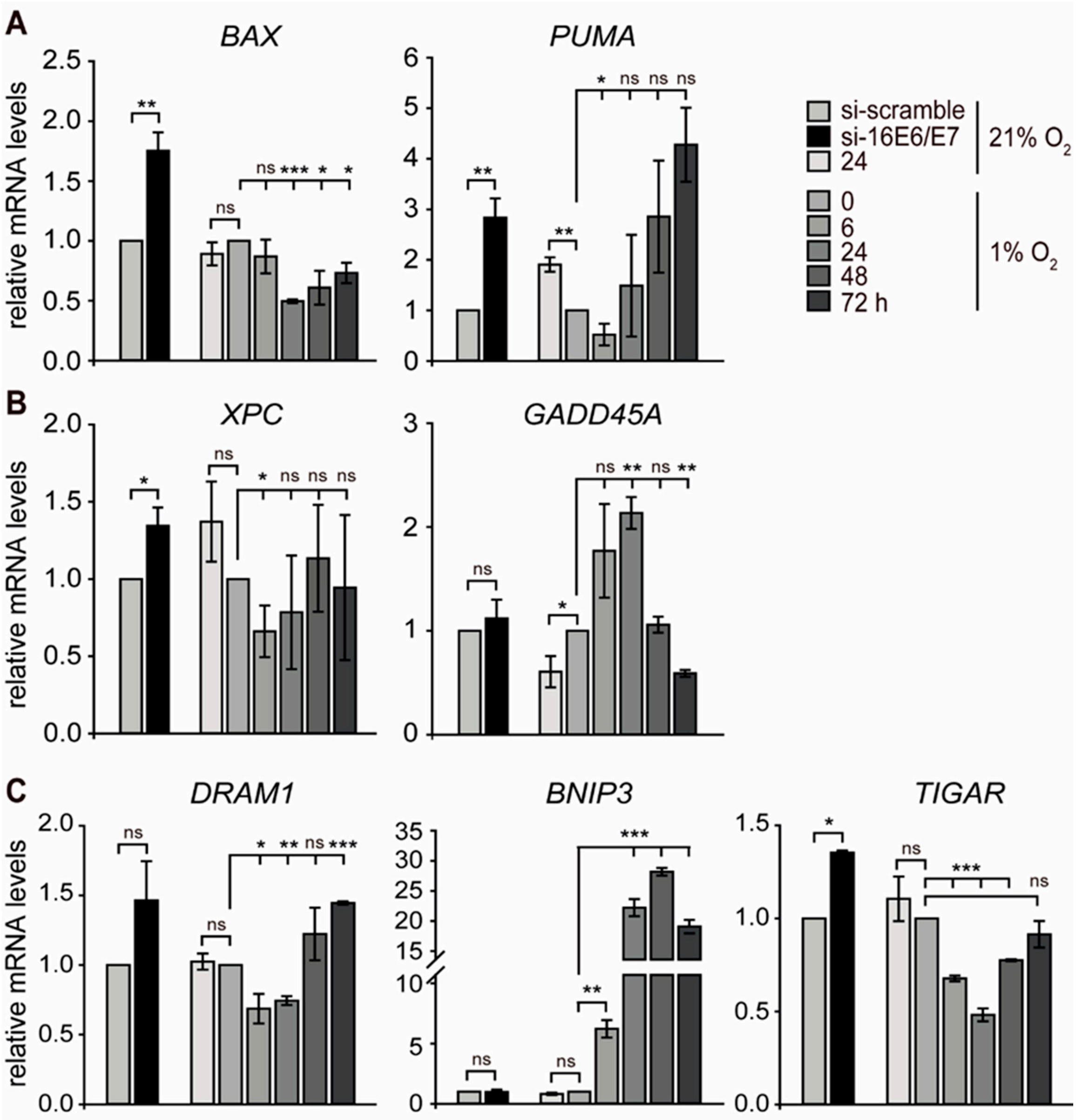

Figure S3. Transcript levels of p53-responsive genes involved in apoptosis, cell cycle arrest/DNA repair, and autophagy in HPV16-positive CaSki cells. HPV16 *E6*/*E7* expression was silenced by RNAi under normoxia in CaSki cells (si-16E6E7), and total RNA was extracted 48 h after transfection. In parallel, cells were cultured for the indicated times under normoxia (21% O_2) or hypoxia (1% O_2), and total RNA was extracted after respective time intervals. qPCR analyses of *BAX* and *PUMA* (**A**); *XPC* and *GADD45A* (**B**); and *DRAM1*, *BNIP3*, and *TIGAR* (**C**) transcripts. The *18S* rRNA served as internal control. Error bars indicate the SD of three independent experiments. The si-scramble group or the 0 h hypoxia value of gene expression was set to 1.0 as a control to calculate significant differences (two-tailed Student's t-test was employed,*** $p < 0.001$; ** $p < 0.01$, * $p < 0.05$ and ns, no significant difference).

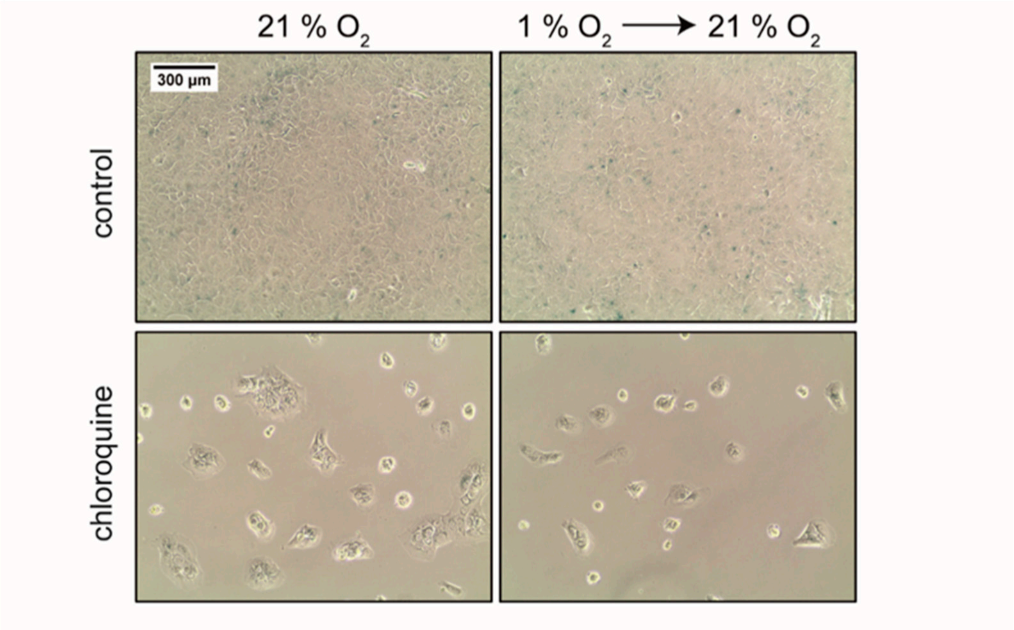

Figure S4. Analyses of cellular senescence upon treatment with autophagy inhibitor CQ. CaSki cells were cultured for 48 h under normoxia (21% O_2) or hypoxia (1% O_2), in either the absence (top) or the presence (bottom) of 50 µM chloroquine (CQ). Subsequently, cells were passaged and cultivated at 21% oxygen for additional 72 h and stained for the senescence marker SA-β-Gal. Scale bar: 300 µm.
